# The impact of screw length on postoperative mucosal thickening in le fort i osteotomy

**DOI:** 10.1007/s00423-026-03969-9

**Published:** 2026-01-16

**Authors:** Sümer Münevveroğlu, Mine Cihan Münevveroğlu, Ceylan Güzel

**Affiliations:** 1 Department of Oral and Maxillofacial Surgery, Faculty of Dentistry, Istanbul Medipol University, Istanbul, Türkiye; 2 Department of Oral and Maxillofacial Surgery, Faculty of Dentistry, Graduate School of Health Sciences, Istanbul Medipol University, Istanbul, Türkiye

**Keywords:** Le fort i osteotomy, Mucosal thickening, Screw length

## Abstract

**Background:**

This study aimed to determine the optimal screw length in Le Fort I osteotomy and to evaluate its specific relationship with postoperative nasal mucosal thickening.

**Materials and methods:**

This retrospective study analyzed 37 patients who underwent Le Fort I osteotomy, either in isolation or in combination with mandibular surgery. Postoperative CT scans were utilized to measure screw lengths and bone widths. Postoperative complications, including maxillary mobility, mucosal thickening, and epiphora due to iatrogenic injury to the nasolacrimal duct, were recorded and analyzed.

**Results:**

The study population consisted of 25 female and 12 male patients, with a mean age of 26.49 ± 6.75 years. Among the 592 screws analyzed, 530 (89.5%) exceeded the optimal length, while only 62 (10.5%) met the recommended criteria. Statistical analysis revealed a significant difference in bone thickness between the piriform and zygomatic buttress regions (*p* < 0.001). No statistically significant difference in bone thickness was found between males and females. However, there was a significant association between excessive screw length and the occurrence of postoperative mucosal thickening (*p* = 0.033).

**Conclusion:**

Accurate screw length selection is crucial for reducing postoperative mucosal thickening. A 5 mm screw length may provide stable fixation while reducing the risk of sinus mucosal thickening, potentially contributing to improved surgical outcomes and patient satisfaction.

## Background

Dentofacial deformities, encompassing various skeletal and dental discrepancies, significantly impact oral function and facial aesthetics [1]. These deformities can lead to difficulties in speech, chewing, and overall self-confidence [2, 3]. Consequently, they have garnered considerable attention within the field of maxillofacial surgery, driving the development of surgical techniques aimed at correcting these conditions. Le Fort I and sagittal split ramus osteotomies are among the most frequently performed procedures for dentofacial deformities [4, 5].

Traditionally, mandibular surgeries were the primary focus in the treatment of dentofacial deformities due to the technical challenges associated with mobilizing the maxilla without compromising blood supply and achieving stable fixation^1^. However, advancements in rigid internal fixation systems within the craniomaxillofacial field have revolutionized the management of these deformities, making Le Fort I osteotomy a safe and effective technique for correcting dentofacial discrepancies, either in isolation or in conjunction with mandibular procedures [6, 7]. Maxillary fixation using 1.5–2 mm L-shaped titanium plates at the piriform and zygomatic buttress regions has become standard practice for achieving stable osteosynthesis. These plates are meticulously contoured and molded to provide optimal stability and promote proper healing [6, 8, 9].

The stability of screw fixation is crucial for the long-term success of Le Fort I osteotomy, as improper length can compromise outcomes. Excessive screw length can compromise the integrity of the fixation, leading to postoperative complications such as screw loosening, maxillary mobility, and mucosal thickening [10–12]. When screws exceed the optimal length, stability may decrease due to excessive engagement with the bone, increasing the risk of loosening and mucosal irritation.It is important to note that emergency screws may be used in situations where conventional screws loosen. Emergency screws may also be utilized to enhance screw stability when the bone in the sinus lateral wall or nasal cavity wall is thin. The optimal screw length for Le Fort I osteotomy fixation remains undefined.

The primary objective of this study is to determine the optimal screw length required for stable fixation in Le Fort I osteotomy and to specifically assess its association with postoperative nasal mucosal thickening. By clarifying this relationship, the study aims to provide practical guidance for screw selection to minimize complications and improve surgical outcomes.

## Materials and methods

This retrospective study included 37 patients who underwent Le Fort I osteotomy with or without concurrent mandibular surgery for the correction of dentofacial deformities. The study was conducted at the Department of Oral and Maxillofacial Surgery, Istanbul Medipol University, between August 2021 and October 2022. The study protocol was approved by the Ethics Committee of Istanbul Medipol University (Decision No: E-10840098-202.3.3.3.02–868) and was conducted in accordance with the Declaration of Helsinki.

Patients were included in the study if they had undergone Le Fort I osteotomy and had available postoperative computed tomography (CT) scans, with a minimum follow-up period of 6 months. Patients were excluded if they had a history of previous maxillary surgery, pre-existing sinus mucosal thickening (to avoid confounding factors), or had undergone alternative maxillary osteotomy techniques other than conventional Le Fort I or High Le Fort I osteotomy.

### Data collection and measurements

**Fig. 1:**
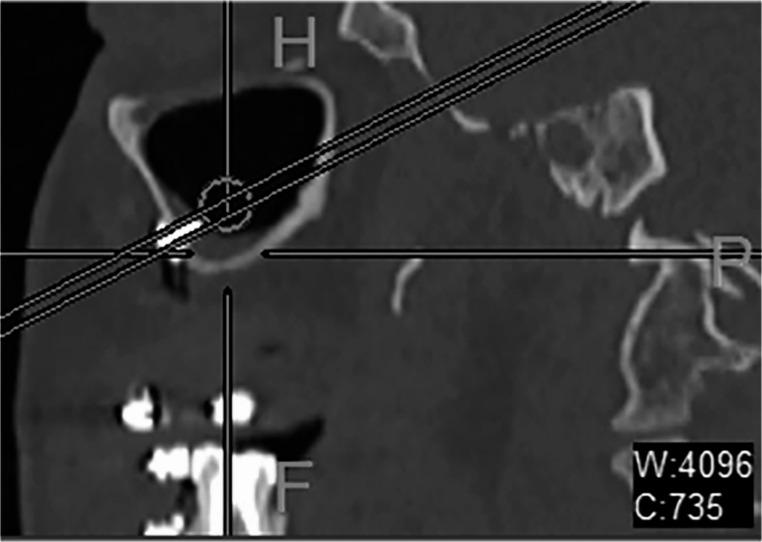
Postoperative CT scan showing measurement of screw length in the piriform aperture and zygomatic buttress

Demographic data, including age and gender, were retrieved from patient records. The placement and dimensions of L-shaped titanium plates (1.5–2 mm) were assessed postoperatively. To determine the recommended screw length, postoperative CT scans were resliced parallel to the long axis of the screws for accurate assessment (Fig. 1).

Bilaterally piriform and zygomatic buttress plates were assessed, and 16 screws were measured in all samples. Measurements were taken for all screws present on these plates.

**Fig. 2:**
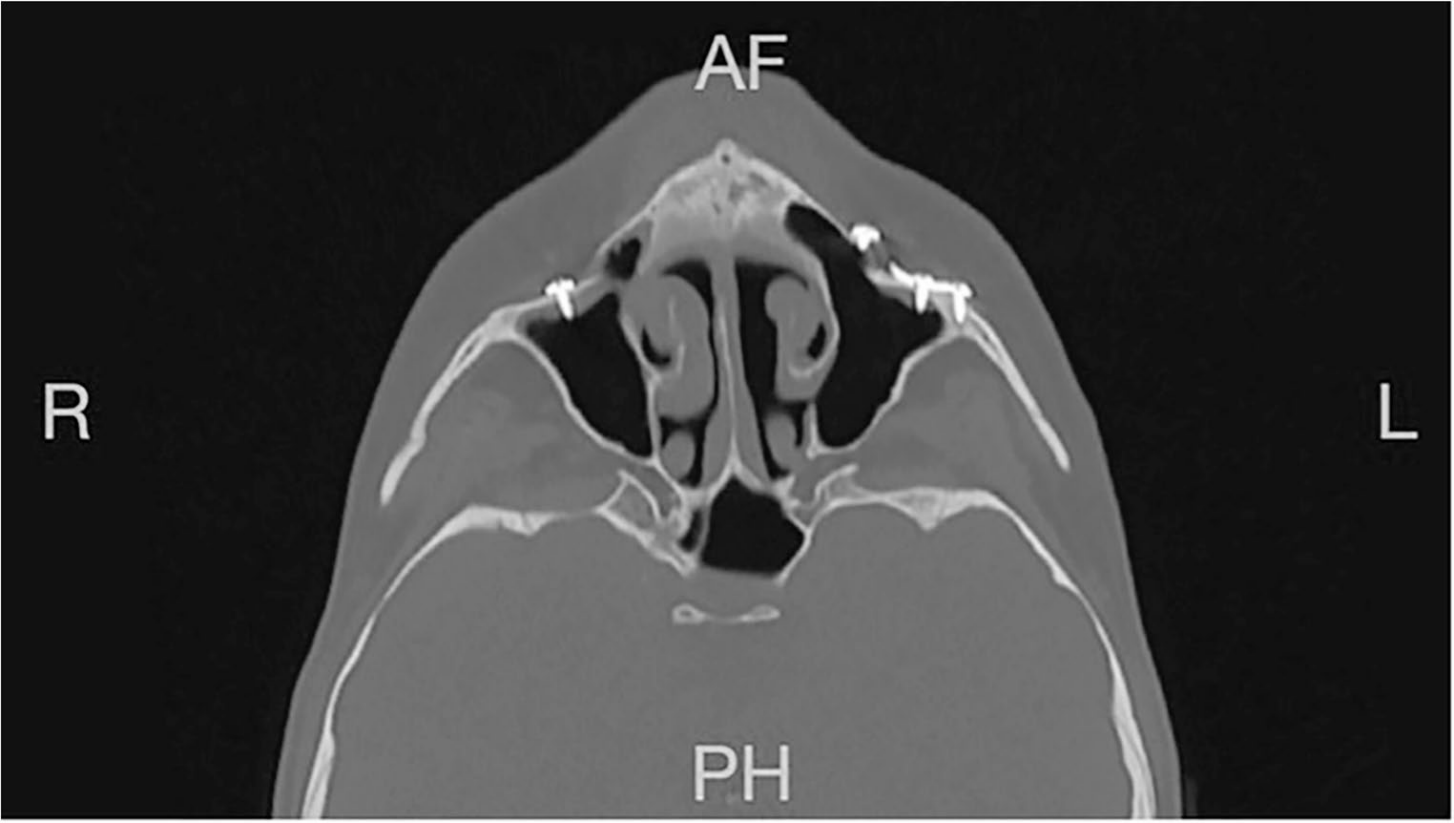
Postoperative axial CT scan showing mucosal thickening in a patient with excessive screw length

The length of each screw was measured, and the recommended ength was calculated by measuring the distance from the screw head to the sinus cavity and nasal area. On postoperative CT scans the presence of mucosal thickening also along with any documented instances of maxillary mobility, epiphora or any other complications (Fig. 2).

### Statistical analysis

Data were analyzed using IBM SPSS Statistics 22 software (IBM SPSS, Turkey). Normality testing was performed using the Shapiro-Wilk test. Differences in bone thickness between male and female patients were analyzed using independent samples t-tests, while paired t-tests were used to compare bone thickness between the piriform aperture and zygomatic buttress regions. The correlation between screw length and mucosal thickening was assessed using Pearson correlation analysis. A p-value < 0.05 was considered statistically significant.

## Results

A total of 37 patients (25 females, 12 males) were included in the study, with a mean age of 26.49 ± 6.75 years. Postoperative computed tomography (CT) scans were analyzed to assess screw length, bone thickness, and postoperative complications.

A total of 592 screws were evaluated across all patients. Among these, 530 screws (89.5%) exceeded the optimal length, while only 62 screws (10.5%) were classified as optimal based on the measured bone thickness. Statistical analysis revealed a significant difference in bone thickness between the piriform and zygomatic buttress regions **(***p* < 0.001**)**, with the piriform region exhibiting greater mean bone thickness (3.45 ± 1.72 mm) compared to the zygomatic buttress region (2.88 ± 1.60 mm). However, no significant difference in bone thickness was observed between male and female patients (*p* > 0.05).

Mucosal thickening was observed in patients whose screws exceeded the optimal length. A statistically significant association was found between excessive screw length and the occurrence of mucosal thickening (*p* = 0.033). However, no statistically significant relationship was identified between **s**crew length and maxillary mobility or epiphora **(***p* > 0.05**)**. One patient developed epiphora due to iatrogenic injury to the nasolacrimal duct, and another patient experienced maxillary mobility, both of whom had screws penetrating beyond the nasal cavity or sinus wall.

**Fig. 3:**
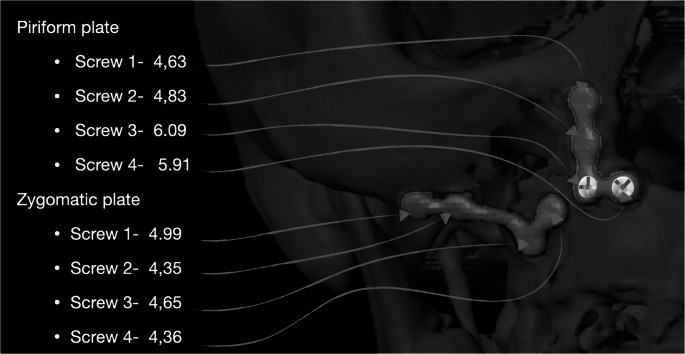
Bone width for the piriform and zygomatic plates

The third and fourth screws of the piriform plate consistently showed greater bone thickness compared to other regions (Fig. 3). This suggests that screw placement should be carefully adjusted based on anatomical variations to optimize stability and minimize complications.

- 89.5% of screws were longer than the optimal length, increasing the risk of mucosal thickening.
- Significant bone thickness difference between the piriform and zygomatic buttress regions (*p* < 0.001).
- Screw length was significantly associated with postoperative mucosal thickening (*p* = 0.033).
- No significant association was found between screw length and maxillary mobility or epiphora (*p* > 0.05).

## Discussion

Dentofacial deformities present significant challenges in the field of oral and maxillofacial surgery, affecting both oral function and facial aesthetics [1]. In Le Fort I osteotomy, titanium plates with 1.5- or 2 mm L-shaped designs are commonly used to stabilize the maxilla at the piriform and zygomatic buttress regions, which are the thickest parts of the bone is [13]. Appropriate screw length selection and accurate screw placement is essential for achieving stable fixation and promoting proper bone healing. However, the ideal screw length in Le Fort I osteotomy has not been clearly defined, and the potential impact of excessive screw lengths on postoperative complications is unknown.

This study highlights the importance of accurate screw length selection in Le Fort I osteotomy, particularly in minimizing postoperative complications such as mucosal thickening. The results indicate that 89.5% of screws used in this study exceeded the optimal length, and a statistically significant association was found between excessive screw length and mucosal thickening (*p* = 0.033). These findings suggest that screws penetrating beyond the bony maxillary wall may cause irritation to the sinus mucosa, leading to chronic inflammation and thickening. Previous studies have described mucosal alterations following Le Fort I osteotomy, but the present study provides quantitative evidence linking screw length with sinus mucosal changes, reinforcing the need for precise preoperative planning and intraoperative verification of screw placement [11, 12]. Our findings are consistent with previous reports demonstrating mucosal alterations following Le Fort I osteotomy [11, 12]. However, unlike prior studies that primarily described mucosal changes qualitatively, the present study provides quantitative evidence directly linking screw length to mucosal thickening.

A significant finding of this study was the variation in bone thickness between the piriform aperture and zygomatic buttress regions. Statistical analysis confirmed that the piriform region exhibited greater mean bone thickness (3.45 ± 1.72 mm) compared to the zygomatic buttress region (2.88 ± 1.60 mm) (*p* < 0.001). This suggests that a standardized screw length may not be appropriate for all fixation sites, as regional differences in bone structure require site-specific adaptations in screw selection. Notably, the third and fourth screws placed in the piriform region had greater bone support, indicating that screw penetration into adjacent structures can be minimized with proper selection. These findings are consistent with previous reports on maxillary bone variability, reinforcing the need for region-specific screw selection [7, 8].

In addition to mucosal thickening, this study examined the relationship between screw length and other postoperative complications, including maxillary mobility and epiphora. No statistically significant association was found between screw length and maxillary mobility (*p* > 0.05), suggesting that fixation stability may not be significantly compromised even if screws slightly exceed the optimal length. However, one case of iatrogenic epiphora was observed due to screw penetration into the nasolacrimal duct, highlighting the potential risks of excessive screw length in superior maxillary regions. This finding is consistent with previous reports documenting nasolacrimal duct obstruction following Le Fort I osteotomy [14, 15], further underscoring the need for careful screw placement, particularly in proximity to the nasal cavity. Based on these results, we recommend the routine use of 5 mm screws for fixation at the piriform and zygomatic buttress regions, as this length provides adequate stability while minimizing the risk of postoperative sinus mucosal complications.

This recommendation aligns with previous studies suggesting that shorter screws are sufficient for stability and may reduce the need for hardware removal due to sinus-related symptoms [10–12].Despite the clinical relevance of these findings, certain limitations must be acknowledged. First, the retrospective study design introduces inherent biases, as data collection relies on existing clinical records and imaging rather than prospective, standardized measurements. Second, the sample size (*n* = 37) is relatively small, potentially limiting the generalizability of the findings. Another limitation is that this study did not evaluate the long-term stability of screw fixation beyond six months, which may affect mucosal remodeling and bone adaptation over time.Future multi-center studies with larger patient cohorts are warranted to validate these results and refine surgical guidelines. Additionally, while CT-based measurements provided precise screw length and bone thickness assessments, bone density variations and individual healing responses were not evaluated, both of which may impact fixation stability and complication rates.

## Conclusion

In conclusion, this study underscores the importance of accurate screw length selection in Le Fort I osteotomy. The findings suggest that a 5 mm screw length appears to provide sufficient stability while minimizing the risk of mucosal thickening and other complications. Additionally, the observed variability in bone thickness across fixation sites highlights the importance of individualized screw selection based on regional anatomical differences. Future research should focus on establishing standardized guidelines for screw length selection that incorporate site-specific bone thickness measurements, thereby further optimizing surgical outcomes and reducing postoperative complications.

## Data Availability

The data supporting the findings of this study are available from the corresponding author upon reasonable request.
